# Glucocorticoid receptor (*NR3C1*) genetic polymorphisms and the outcomes of sudden sensorineural hearing loss

**DOI:** 10.1186/s40463-022-00601-w

**Published:** 2023-02-12

**Authors:** Chen-Yu Chien, Shu-Yu Tai, Kuan-Hui Li, Hua-Ling Yang, Ling-Feng Wang, Kuen-Yao Ho, Ning-Chia Chang

**Affiliations:** 1 Department of Otorhinolaryngology, Kaohsiung Medical University Hospital, Kaohsiung Medical University, No. 100, Tzyou 1st Road, Kaohsiung, 807 Taiwan; 2 Department of Family Medicine, School of Medicine, College of Medicine, Kaohsiung Medical University, Kaohsiung, Taiwan; 3 Division of Hepatobiliary and Pancreatic Medicine, Department of Internal Medicine, Kaohsiung Medical University Hospital, Kaohsiung Medical University, Kaohsiung, Taiwan; 4 Department of Otorhinolaryngology, Kaohsiung Municipal Siaogang Hospital, Kaohsiung, Taiwan; 5 Department of Family Medicine, Kaohsiung Municipal Ta-Tung Hospital, Kaohsiung, Taiwan; 6 Department of Otorhinolaryngology, School of Medicine, College of Medicine, Kaohsiung Medical University, Kaohsiung, Taiwan; 7 Department of Family Medicine, Kaohsiung Medical University Hospital, Kaohsiung Medical University, Kaohsiung, Taiwan

**Keywords:** Nuclear receptor subfamily 3 Group C member 1 (*NR3C1*), Genetic polymorphism, Outcomes, Steroid receptor gene, sudden sensorineural hearing loss

## Abstract

**Background:**

The glucocorticoid receptor gene (*NR3C1*) encodes the receptor to which cortisol and other glucocorticoids bind. Steroids in either oral, intratympanic, or intravascular forms are the treatment of choice for sudden sensorineural hearing loss (SSNHL), but the outcome varies. The outcomes of SSNHL have been investigated for related factors, including age, initial hearing loss severity and pattern, vertigo, genetic variations, and the time between onset and treatment. The objective of the present study was to analyze the association of genetic polymorphisms of *NR3C1* with the outcomes of SSNHL.

**Materials and methods:**

We conducted a comparison study of 93 cases with a poor outcome (control) and 100 cases with a good outcome (case) in SSNHL patients. Six single nucleotide polymorphisms (SNPs) were selected. The genotypes were determined using TaqMan technology.

**Results:**

The heterozygous AT genotype of rs17100289 was associated with a poor outcome in comparison with the major homozygous AA genotype after adjustments for age and sex (OR = 0.50; 95% CI 0.26–0.95; *P* = 0.035) in SSNHL patients. The CT genotype of rs4912912 was also associated with a poor outcome compared with the major homozygous TT genotype after the adjustments (OR = 0.47; 95% CI 0.24–0.92; *P* = 0.026).

**Conclusion:**

These results suggest that *NR3C1* genetic polymorphisms may influence the outcomes of SSNHL.

**Graphical Abstract:**

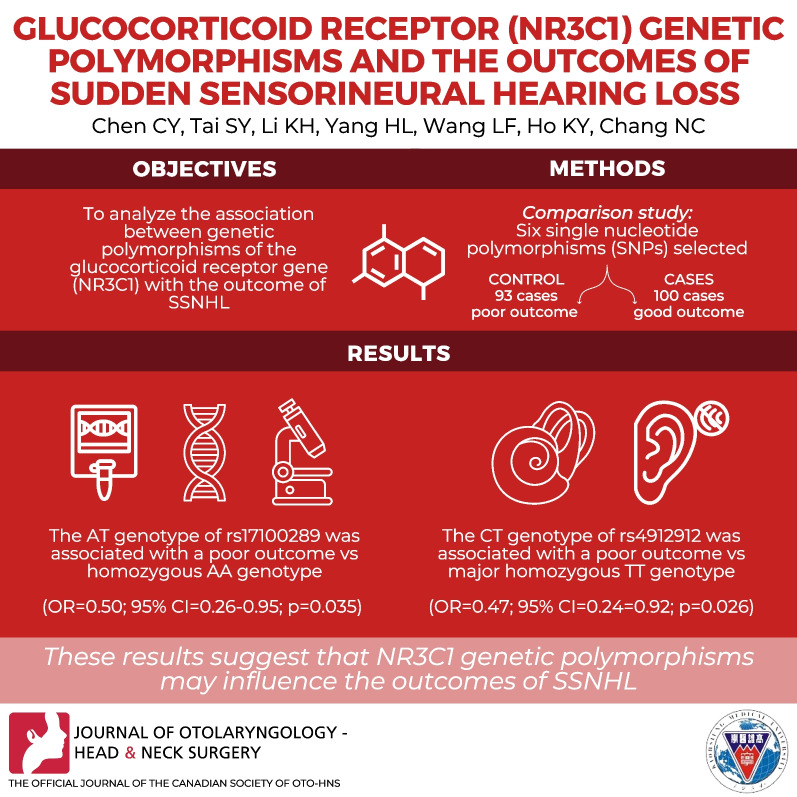

## Introduction

Sudden sensorineural hearing loss (SSNHL) is defined as a loss of at least 30 dB in three contiguous frequencies over 3 days or less [1]. The estimated incidence is between 5 and 20 per 100,000 persons per year in the United States [1]. The incidence rates per a population of 100,000 in Taiwan are 8.85 for men and 7.79 for women [2]. The detailed etiology and pathogenesis of SSNHL are still unclear. Potential causes may include viral infection, vascular disease, intracochlear membrane rupture, autoimmunity, and genetic factors. [3]. Histopathologic studies of SSNHL revealed changes in the cochlea, including atrophy of the organ of Corti and loss of cochlear neurons, which was the main finding in viral Inflammation [4]. However, there is no consensus, and the etiology and pathogenesis of SSNHL remain controversial.

Merchant et al. [5] revealed that a final common pathway for several possible etiologies of sudden sensorineural hearing loss may be the result of pathologic activation of cellular stress pathways involving nuclear factor kappa B (NF-kB) within the cochlea. Cortisol acts in the regulation of other glucocorticoid-responsive genes and is involved in interactions between the cortisol-glucocorticoid receptor complex and other transcription factors, such as NF-kB. The cortisol-glucocorticoid receptor complex also physically interacts with NF-kB to block its transcriptional activity leading to the anti-inflammatory effect [6].

Several factors related to the outcomes of SSNHL, including age, initial hearing loss severity and pattern, vertigo, and the time between onset and treatment, have been investigated [7–9]. Steroids (glucocorticoids) in either oral, intratympanic, or intravascular forms are the treatment of choice for SSNHL patients, but the outcome varies [7, 10]. The efficacy of glucocorticoids (GCs) in alleviating inflammatory disorders results from the pleiotropic effects of the glucocorticoid receptor (GR) on multiple signaling pathways [6]. The distribution of GRs in the cochlea appears to be subsite specific. GRs are expressed at highest concentrations with in the organ of Corti, followed by the spiral ganglion neurons (SGNs), and are present in lower concentration within the stria vascularis and fibrocytes of the lateral cochlear wall [11]. In an animal study, the authors found intense nuclear immunofluorescence at basal and apical SGN regions after GC administrated either via local or systemic route, which was greater than control tissue. And the GR activation of the SGNs was equivalent in both administration regimens [12]. The above findings indicate that GCs may bind to the nuclear GRs in the inner ear to perform the anti-inflammatory effect in SSNHL.

GR is a member of the steroid hormone receptor family of proteins [13]. GR binds with high affinity to cortisol in the cell, and the cortisol-GR complex inhibits inflammation through direct and indirect genomic effects and nongenomic mechanisms[6]. GR is known as nuclear receptor subfamily 3 Group C member 1 (*NR3C1*). The *NR3C1* gene is located on chromosome 5q31–32. Variation in the structure and the expression of the gene generates diversity in glucocorticoid signaling [14]. However, there is little published literature about the relationship between the corticosteroid receptor gene and the outcomes of SSNHL. Kitoh et al. reported a marginal correlation between the single nucleotide polymorphism (SNP) of the *NR3C1* gene (rs4912910) and steroid treatment outcomes in SSNHL patients [15]. Genetic polymorphisms of the *NR3C1* gene might alter the affinity of GR to corticosteroids, affect the anti-inflammatory effect, and lead to a poorer outcome in SSNHL.

There is no doubt that the evaluation of genetic polymorphisms in SSNHL is important because identifying patients with genotypes with a poor outcome may help us develop optimal treatment strategies. In the present study, we hypothesize that the SNPs of the GR gene (i.e., the *NR3C1* gene) may be crucial for the outcomes of SSNHL. The goal of this study was to understand the role of *NR3C1* genetic polymorphisms in the outcomes of SSNHL after steroid treatment in Taiwan.

## Materials and methods

### Study population

In this prospective study, we randomly recruited participants from SSNHL patients diagnosed and treated in an academic medical center (Kaohsiung Medical University Hospital). The diagnostic criteria of SSNHL were sensorineural hearing loss of at least 30 dB in 3 contiguous frequencies using a pure tone audiogram with an onset within 3 days [2]. Patients who were traditionally considered to have a better outcome were included in the present study. These better prognostic factors included age younger than 50 years, no vertigo, initial hearing loss < 90 dBHL, and less than 7 days from onset to treatment [7–9].

All the patients were hospitalized for one week. They received prednisolone orally (1 mg/kg for 2 days), which was decreased over the next 2 weeks. During hospitalization, all the patients received audiometric tests, including pure tone audiometry (PTA), auditory brainstem-evoked responses, and magnetic resonance imaging (to exclude acoustic neuroma). After discharge, the PTA changes were monitored for at least six weeks to determine the outcome of each patient.

To investigate the factors associated with the efficacy of steroid treatment for SSNHL, the patients were separated into two groups based on outcomes. The classification of good and poor outcomes was based on the recommendations of the “Clinical Practice Guideline: Sudden Hearing Loss” from the American Academy of Otolaryngology-Head and Neck Surgery (AAO-HNS) in 2012[16]. A complete recovery requires hearing return to within 10 dB HL of the unaffected ear and recovery of word recognition sore (WRS) to within 5% to 10% of the unaffected ear. Anything less than a 10 dB HL improvement should be classified as no recovery. Partial recovery defined in 2 ways (clinically meaningful recovery/ not meaningful recovery) based on whether or not the degree of initial hearing loss after the event of SSNHL rendered the ear nonserviceableb (PTA > 50 dB or WRS < 50%). The patients with “complete recovery” and “meaningful partial recovery” were classified into the good outcome (case) group. The patients with “not meaningful partial recovery” and “no recovery” were categorized into the poor outcome (control) group.

## Genetic analysis

### SNP selection

We selected all SNPs of *NR3C1* from the released 2.0 phase II data of the HapMap Project [17] using the Tagger Pairwise method [18]. The SNPs were chosen according to the following criteria: r^2^ was 0.8 or greater and the minor allele frequency (MAF) was more than 25% in the Han Chinese population. Six SNPs met the above criteria and were selected for genotyping: rs6877893, rs4912912, rs17100289, rs12653301, rs4912650, and rs9324924 of the *NR3C1* gene.

### Genotyping

Genomic DNA was extracted from peripheral blood before treatment using a standard method. Genotyping was performed using TaqMan technology (7500 Real Time PCR System, Applied Biosystems, Foster City, CA, USA), and reactions were performed in 96-well microplates in ABI 9700 thermal cyclers (Applied Biosystems, Foster City, CA, USA). Fluorescence was measured using an ABI 7500 Real-Time PCR System and analyzed with System SDS software version 1.2.3. Each subject was typed for all SNPs.

### Statistical analysis

Continuous variables were analyzed using independent t-tests, and the results are presented as the means ± SD. The allele frequency was obtained using direct gene counting. The mixture Hardy–Weinberg equilibrium (mHWE) was examined in the participants[19]. The effect of the minor allele of each SNP was examined in dominant and recessive genetic models. Multivariate logistic regression analysis was performed to obtain the age- and sex-adjusted odds ratio (OR) and 95% confidence interval (95% CI) while assessing the genetic effects. All statistical operations were performed using JMP software version 13.0 (SAS Institute Inc., Cary City, NC, USA) for Windows. Two-tailed *P* values < 0.05 were considered significant.

## Results

### Participants

A total of 193 SSNHL patients were included in the present study. In the poor outcome group, there were 93 patients, including 60 (64.5%) males and 33 (35.5%) females. In the good outcome group, there were 100 patients, including 64 (64.0%) males and 36 (36.0%) females. The characteristics of SSNHL are summarized in Table 1. The mean age of the 193 participants was 38.8 ± 10.8 years. The mean ages were 38.8 ± 10.9 years and 38.9 ± 10.7 years in the poor outcome and good outcome groups, respectively. The male to female ratio was 1.8:1 in the 193 SSNHL patients. The age and sex characteristics were not significantly different between the poor and good outcome groups.

**Table 1:** Demographics of subjects in the poor and good outcomes groups

Outcomes	Poor (n = 93)	Good (n = 100)	*P* value
Age (mean ± SD, y/o)	38.8 ± 10.9	38.9 ± 10.7	0.9304^1^
*Sex (n, %)*			
Male	60(64.5)	64(64.0)	0.9404^2^
Female	33(35.5)	36(36.0)	
*Hearing loss type*			
High	18(19.4)	10(10.0)	0.1556^2^
Flat	64(68.8)	74(74.0)	
Total	11(11.8)	16(16.0)	
*PTA (mean ± SD, dB)*			
Pre-treatment	68.83 ± 24.03	62.71 ± 21.00	0.0605^1^
Post-treatment	56.08 ± 20.56	28.43 ± 16.84	< 0.0001^*1^
Change in hearing level	6.63 ± 12.43	40.40 ± 18.94	< 0.0001^*1^
*SRT (mean ± SD, dB)*			
Pre-treatment	64.38 ± 25.52	56.75 ± 29.31	0.1174^1^
Post-treatment	52.61 ± 26.28	37.15 ± 27.44	0.0013^*1^
*SDS (mean ± SD, %)*			
Pre-treatment	55.39 ± 36.17	62.65 ± 39.00	0.2732^1^
Post-treatment	63.22 ± 37.46	82.52 ± 30.03	0.0013^*1^

^1^ Independent t-test

^2^ χ^2^ test

^*^P < 0.05

Pure-tone average (PTA) by average of pure-tone hearing threshold by air conduction at 0.5, 1, 2, and 3 kHz

Abbreviations: pure-tone average (PTA), speech discrimination score (SDS), speech reception threshold (SRT)

### Genetic analysis

There were significant differences in the distributions of the genotypes of *NR3C1* rs17100289 (χ^2^ test, *P* = 0.003) and rs4912912 (χ^2^ test, *P* = 0.030) between the poor and good outcome groups. The AT genotype of rs17100289 was associated with a poor outcome in comparison with the AA genotype after adjustments for age and sex (OR = 0.50; 95% CI 0.26–0.95; *P* = 0.035). The CT genotype of rs4912912 was also associated with a poor outcome compared with the TT genotype after adjustments (OR = 0.47; 95% CI 0.24–0.92; *P* = 0.026). However, the genotypic and allelic distributions showed no significant difference between the groups for the other SNPs (rs12653301, rs4912650, rs6877893, and rs9324924). The results of allele and genotype analyses are presented in Table 2.

**Table 2:** Associations of six single nucleotide polymorphisms (SNPs) of the *NR3C1* gene with sudden sensorineural hearing loss (SSNHL)

SNP	Overall, n (%)		*P* value^1^	Adjusted OR (95% CI)^2^	*P* value^3^
	Poor	Good			
rs12653301					
*Genotypes*					
GG	31 (33)	34 (34)	0.967	1.00^4^	
AG	42 (45)	46 (46)		1.00 (0.52–1.9)	0.999
AA	20 (22)	20 (20)		1.91(0.41–2.01)	0.822
*Alleles*					
G	104 (56)	114 (57)	0.830	1.00^4^	
A	82 (44)	86 (43)		0.958 (0.64–1.43)	0.834
rs17100289					
*Genotypes*					
AA	28 (30)	37 (37)	0.003^*^	1.00^4^	
AT	54 (58)	36 (36)		0.50 (0.26–0.95)	0.035^*^
TT	11 (12)	27 (27)		1.84 (0.79–4.46)	0.159
*Alleles*					
A	110 (59)	110 (55)	0.412	1.00^4^	
T	76 (41)	90 (45)		1.18 (0.79–1.78)	0.416
rs4912650					
*Genotypes*					
TT	36 (39)	40 (40)	0.917	1.00^4^	
GT	42 (45)	46 (46)		0.99 (0.53–1.83)	0.963
GG	15 (16)	14 (14)		0.84 (0.35–1.98)	0.689
*Alleles*					
T	114 (61)	126 (63)	0.729	1.00^4^	
G	72 (39)	74 (37)		0.93 (0.62–1.40)	0.728
rs4912912					
*Genotypes*					
TT	24 (26)	36 (38)	0.030^*^	1.00^4^	
CT	53 (57)	38 (38)		0.47 (0.24–0.92)	0.026^*^
CC	16 (17)	26 (26)		1.09 (0.49–2.47)	0.836
*Alleles*					
T	101 (54)	110 (55)	0.890	1.00^4^	
C	85 (46)	90 (45)		0.97 (0.65–1.45)	0.891
rs6877893					
*Genotypes*					
AA	54 (58)	57 (57)	0.988	1.00^4^	
AG	31 (33)	34 (34)		1.04 (0.56–1.92)	0.903
GG	8 (9)	9 (9)		1.07 (0.38–3.03)	0.904
*Alleles*					
A	139 (75)	148 (74)	0.869	1.00^4^	
G	47 (25)	52 (26)		1.04 (0.66–1.64)	0.871
rs9324924					
*Genotypes*					
GG	32 (34)	32 (32)	0.931	1.00^4^	
GT	49 (53)	54 (54)		1.11 (0.59–2.07)	0.754
TT	12 (13)	14 (14)		1.17 (0.47–2.98)	0.732
*Alleles*					
G	113 (61)	118 (59)	0.726	1.00^4^	
T	73 (39)	82 (41)		1.08 (0.72–1.62)	0.717

^1^χ^2^ test

^2^Adjusted for age and sex

^3^Logistic regression

^4^Reference group

^*^P < 0.05

The hereditary models (dominant and recessive) were analyzed in each SNP. A significant result was observed from the recessive model for *NR3C1* rs17100289, which yielded an adjusted OR of 2.76 (95% CI 1.31–6.17, *P* = 0.007) for the TT genotype compared with the AA + AT genotypes after adjustment for age and sex. Participants who carried the TT homozygote of *NR3C1* rs17100289 were associated with a better outcome of SSNHL. However, no significant association of the hereditary model with the outcomes of SSNHL was found in the other five SNPs (rs12653301, rs4912650, rs4912912, rs6877893, and rs9324924). The hereditary models are summarized in Table 3.

**Table 3:** Dominant and recessive hereditary models

SNP	Overall, n (%)		*P* value^1^	Adjusted OR (95% CI)^2^	*P* value^3^
	Poor	Good			
rs12653301					
*Genotypes*					
GG	31 (33)	34 (34)	0.922	1.00^4^	
AG + AA	62 (67)	66 (66)		0.97 (0.53–1.77)	0.927
GG + AG	73 (78)	80 (80)	0.797	1.00^4^	
AA	20 (22)	20 (20)		0.91 (0.45–1.84)	0.799
rs17100289					
*Genotypes*					
AA	28 (30)	37 (37)	0.311	1.00^4^	
AT + TT	65 (70)	63 (63)		0.73 (0.39–1.33)	0.301
AA + AT	82 (88)	73 (73)	0.008^*^	1.00^4^	
TT	11 (12)	27 (27)		2.76 (1.31–6.17)	0.007^*^
rs4912650					
*Genotypes*					
TT	36 (39)	40 (40)	0.855	1.00^4^	
GT + GG	57 (61)	60 (60)		0.95 (0.53–1.69)	0.854
TT + GT	78 (84)	86 (86)	0.679	1.00^4^	
GG	15 (16)	14 (14)		0.85 (0.381.87)	0.678
rs4912912					
*Genotypes*					
TT	24 (26)	36 (36)	0.126	1.00^4^	
CT + CC	69 (74)	64 (64)		0.62 (0.33–1.14)	0.124
TT + CT	77 (83)	74 (74)	0.134	1.00^4^	
CC	16 (17)	26 (26)		1.70 (0.85–3.49)	0.134
rs6877893					
*Genotypes*					
AA	54 (58)	57 (57)	0.881	1.00^4^	
AG + GG	39 (42)	43 (43)		1.04 (0.59–1.85)	0.882
AA + AG	85 (91)	91 (91)	0.922	1.00^4^	
GG	8 (9)	9 (9)		1.05 (0.38–2.92)	0.923
rs9324924					
*Genotypes*					
GG	32 (34)	32 (31)	0.723	1.00^4^	
GT + TT	61 (66)	68 (68)		1.12 (0.61–2.05)	0.715
GG + GT	81 (87)	86 (86)	0.823	1.00^4^	
TT	12 (13)	14 (14)		1.10 (0.48–2.59)	0.816

^1^χ^2^ test

^2^Adjusted for age and sex

^3^Logistic regression

^4^Reference group

^*^P < 0.05

## Discussion

This study investigated the relationship between six SNPs of the *NR3C1* gene and the outcomes of SSNHL in a Taiwanese population and found that *NR3C1* has an evident genetic effect on the outcomes of SSNHL. The significant result stemmed from the rs17100289 polymorphisms of the *NR3C1* gene, highlighting the role this rare TT genotype of rs17100289 has in the protection against SSNHL in Taiwan. Patients with the CT genotype of rs4912912 had a higher risk of a poor outcome than those with the major homozygous genotype TT. We found no significant difference between the poor and good prognostic groups at SNPs rs12653301, rs4912650, rs6877893, and rs9324924 in SSNHL. This investigation is the first report of the potential contribution specifically by *NR3C1* genetic variants to the outcomes of SSNHL.

Furthermore, our recessive models yielded a significant adjusted OR of 2.76 (*P* = 0.007) for TT versus AA + AT of rs17100289 after adjustment for age and sex. This result revealed a tendency for TT to have a better prognostic effect on SSNHL than the AA + AT genotype. The TT allele of rs17100289 seems to be a protective genotype with a recessive effect on SSNHL in Taiwan. The insignificant result of the minor homozygous TT compared with the major homozygous AA genotypes of rs17100289 in the genotype analysis might be caused by the small number of TT carriers in this study population. SNP rs17100289 has not been previously reported to be associated with hearing impairment.

The outcomes of SSNHL remain a controversial issue and is usually assessed by analyses of factors that correlate with hearing improvement. The results were inconsistent due to the various therapeutic strategies and multiple outcome definitions for recovery. The outcome of SSNHL has been investigated for its relation to several factors, including age, initial hearing loss severity and pattern, vertigo, underlying diseases, and the duration between onset and treatment [9]. Steroids have been used to treat sudden sensorineural hearing loss for a long time, but the results varied even in patients with the same condition. We have different steroid receptor genotypes and phenotypes; perhaps this is the reason why we have varied results even with the same treatment. However, there are few studies about the relationship between the genetic variations of steroid receptors and the outcomes of SSNHL.

The GR gene (*NR3C1*) has a three-domain structure: an amino-terminal transactivation domain, a DNA-binding domain, and carboxy-terminal ligand binding [6, 20]. The carboxy-terminal ligand binding domain contains specific steroid and heat shock protein binding sites [6, 21]. Previous research revealed that heat shock protein 70 gene polymorphisms influence the risk of SSNHL in Taiwan [22]. During the unliganded form of the GR, it is located in the cytoplasm in a large complex form of protein. After binding to cortisol, it dissociates, and a conformational change occurs. Then, the GR translocates to the nucleus, where it acts as a transcription factor to regulate the transcription of the GR gene by several mechanisms [23].

GR gene polymorphism is a possible factor that influences GC sensitivity. A number of polymorphisms in the GR gene are known. A few of these polymorphisms are functionally relevant. ER22/23EK (rs6189 and rs6190), N363S (rs6195), and BclI (rs41423247) are the three most commonly investigated GR gene polymorphisms [20]. The ER22/23EK polymorphism and relative GC resistance have shown diminished transactivational activity. The N363S polymorphism was reported to be associated with enhanced sensitivity to GCs. The BclI polymorphism is also associated with increased GC sensitivity [20].

The strength of this study is that the results give us a light to the precision medicine in the treatment of SSNHL. Based on the genetic differences, we might tailor the treatment strategy for each SSNHL patient to get the best outcome in the future. There are several limitations in the present study. The major limitation of this study is that it only included subjects in Eastern Asia. Further studies among different ethnic groups are needed to further generalize our results. The second limitation is the small sample size in the present study. The results of the present study would be more evident if the sample size enlarged. Another limitation is that only the patients traditionally considered to have better outcomes were included in the present study. To include all patients without predicting the outcomes might make the results more convincing. In addition, further functional analyses of the SNPs in the *NR3C1* gene and their relationship with SSNHL are warranted.

## Conclusions

The results of this study support the finding that genetic polymorphisms of the *NR3C1* gene are associated with the outcomes of SSNHL after steroid treatment in a Taiwanese population.

## Data Availability

Not applicable.
